# ‘Are they just putting up with me’? How diversity approaches impact LGBTQ+ employees' sense of being tolerated at work

**DOI:** 10.1111/bjso.70006

**Published:** 2025-08-01

**Authors:** Kshitij Mor, Seval Gündemir, Jojanneke van der Toorn

**Affiliations:** ^1^ Organizational Behavior Group, Faculty of Behavioral and Social Sciences Utrecht University Utrecht the Netherlands; ^2^ Rotterdam School of Management Erasmus University Rotterdam Rotterdam the Netherlands; ^3^ Institute of Psychology Leiden University Leiden the Netherlands

**Keywords:** diversity approach, leadership, LGBTQ+, tolerance

## Abstract

This research investigates whether and how workplace diversity approaches—identity‐conscious versus identity‐blind—are associated with LGBTQ+ employees' perceptions of tolerance. Whilst tolerance is widely regarded as an important virtue for the harmonious functioning of diverse societies, it can inadvertently harm minoritized individuals. In workplace settings, perceptions of tolerance may hinder the benefits of diversity by discouraging minoritized employees from sharing their perspectives and prompting individuals with relatively concealable stigmas, such as LGBTQ+ employees, to conceal their identities. Across two studies (*n* = 907), we examine the conditions under which tolerance perceptions may arise. Study 1 explores LGBTQ+ prospective employees' anticipated tolerance in organizations with identity‐blind versus identity‐conscious mission statements. Study 2 examines LGBTQ+ employees' workplace experiences, focussing on how organizational and leadership diversity approaches are related to perceptions of tolerance. Findings reveal that relatively identity‐blind approaches are associated with increased feelings of being tolerated. Moreover, identity‐conscious leadership strategies, when coupled with identity‐conscious organizational approaches, further diminish perceptions of being merely tolerated. Our findings underscore an un‐intended correlate of identity‐blind diversity approaches, which may perpetuate tolerance‐focussed climates and indirectly undermine inclusion for LGBTQ+ employees.

## INTRODUCTION

The increasing diversity in the workplace has brought critical conversations to the forefront about how to navigate differences in identity, beliefs and values. In organizational contexts, these conversations are often shaped within broader perspectives on how diversity should be managed. Diversity approaches are prescriptive frameworks that guide individuals' and organizations' engagement with social group differences, such as race, gender and sexual orientation (Wu & Apfelbaum, 2024) and are widely reflected in organizational communication (Kirby et al., 2023).

These approaches can be broadly divided into two categories: the *conscious* approach, which values and celebrates the differences between social groups, and the *blind* approach, which downplays social group differences in favour of individualistic views or an overarching emphasis on group membership. Whilst employees from minoritized groups tend to respond more positively to conscious rather than blind approaches (Gündemir et al., 2019; Mor et al., 2024), the broader literature presents a nuanced view of the impact of these approaches, with effects on belonging, organizational attractiveness and other outcomes depending on conceptual and contextual contingencies (e.g., Kirby et al., 2021; Leslie et al., 2020; Martin, 2023). Given the wide prevalence and substantial impact of these approaches, scholarly interest in understanding which outcomes they influence, for whom and under what conditions, remains high (Kirby et al., 2023). Intriguingly, studies on the impact of diversity approaches often focus on whether these initiatives are beneficial or harmful for certain groups, with outcomes of interest frequently framed in strongly valenced terms (e.g., Plaut et al., 2009, 2018).

Here, we advance this past work by examining the role of diversity approaches in shaping a potentially critical yet understudied outcome, namely, employees’ sense of being tolerated, whose valence is often ambiguous rather than clearly positive or negative. The Cambridge Dictionary (n.d.) defines tolerance as ‘*willingness to accept behavior and beliefs that are different from your own, even if you disagree with or disapprove of them*.’ In popular discourse, tolerance is typically regarded as a virtue that promotes openness and respect (Cohen, 2004; Oberdiek, 2001; Verkuyten et al., 2023). However, scholarly discussions have highlighted the more ambivalent nature of tolerance, pointing to how it can reinforce power imbalances and contribute to feelings of marginalization amongst those who are merely ‘put up with’ by dominant groups (Adelman et al., 2023; Cvetkovska et al., 2020, 2021; Verkuyten et al., 2020). Rather than signalling acceptance or equality, being tolerated can be experienced as conditional and sometimes even condescending. From this perspective, tolerance occupies a conceptual space between exclusion and genuine inclusion. This ambivalence renders it a particularly complex and meaningful perception, whose examination provides novel insights into the effects of diversity approaches that go beyond straightforwardly positive or negative outcomes. Whilst research has begun to define tolerance and examine how it is experienced by minoritized individuals and its consequences (Adelman et al., 2023, 2024; Cvetkovska et al., 2021; Verkuyten et al., 2019), relatively little is known about the contextual or organizational factors that elicit feelings of being tolerated. In the present study, we aim to address these gaps by exploring the relationship between diversity approaches and perceptions of tolerance, focussing on an understudied population: LGBTQ+ employees.

Below, we begin by summarizing diversity approaches, how they are understood in the literature and why it is important to focus on LGBTQ+ employees’ experiences in this context. We then review the academic literature on tolerance, including the scholarly definition guiding this study. We further examine the relevance of perceived tolerance in the workplace, before outlining key organizational and interpersonal factors hypothesized to shape these perceptions. In this final section of our theory, we identify and discuss several additional factors that may moderate or attenuate how LGBTQ+ employees respond to diversity approaches. In addition to considering multiple group memberships (e.g., transgender identity and race), we also explore the role of diversity messages communicated by key organizational actors, that is, leaders, alongside broader organizational statements. This allows us to unpack unique dynamics across different dimensions that may influence LGBTQ+ employees' workplace experiences.

### Navigating diversity in the workplace through diversity approaches

The diversity approach adopted by an organization often serves as a key indicator of its diversity climate (Leslie & Flynn, 2022; Li et al., 2019; Mor Barak et al., 2016). In social and organizational psychology, diversity approaches are typically understood through two variants: identity‐blindness and identity‐consciousness. An identity‐blind approach minimizes demographic differences, emphasizing either a shared identity or individual uniqueness. This approach is grounded in the belief that focussing on demographic differences may inadvertently increase stereotyping and prejudice. In contrast, an identity‐conscious approach values and celebrates demographic differences, recognizing that individuals bring unique experiences and perspectives shaped by their identities, which influence how they contribute to and experience the workplace (Plaut et al., 2009, 2018; Rattan & Ambady, 2013).^1^ Despite variations in methods and specifics, the overarching goal of these diversity approaches is to foster a work environment where diverse groups can collaborate effectively and harmoniously.

Whilst research on diversity approaches has traditionally centred on more visible identities, such as race and gender, recent scholarship has begun to focus on minoritized groups with concealable identities, including LGBTQ+ employees. Examining the experiences of LGBTQ+ individuals in this context is both theoretically and socially significant, as the concealable nature of their identities introduces unique challenges. These challenges differ in important ways from those faced by more visible groups such as racial minorities and women who have historically been the primary focus of diversity scholarship (Gündemir et al., 2019). Emerging findings suggest that identity‐conscious messaging is associated with greater psychological safety, authenticity and a sense of belonging amongst LGBTQ+ employees (Kirby, Barreto, et al., 2023; Mor et al., 2024). These environments make it easier for individuals to express their identities without fear of stigma or marginalization. Whilst studies have linked identity‐blindness to negative outcomes such as increased prejudice (Plaut et al., 2018), it is important to recognize that these approaches are often well‐intentioned strategies for managing diversity. Given that they typically do not involve overtly exclusionary rhetoric, their effects may be more nuanced. Rather than resulting in clear rejection, identity‐blind environments may lead to more ambiguous experiences, neither fully inclusive nor explicitly hostile.

To better understand this in‐between space, we propose that the concept of tolerance may help explain the dynamics at play. In contexts where identity is neither explicitly acknowledged nor outright dismissed, employees may feel merely tolerated. To explore this further, the following section reviews the literature on tolerance and outlines the working definition adopted in this study.

### Defining tolerance and understanding its impact on minoritized individuals

Tolerance is often viewed as a virtue almost akin to acceptance and respect. Some scholars define it as openness to other cultures and a generalized positive attitude towards differences (Allport, 1954; Hjerm et al., 2020). Some scholars propose a respect‐based conception of tolerance, which views groups as moral equals, who despite differences operate from a shared framework of equal status for all (Forst, 2018). Others, however, argue that disapproval and negative attitudes are inherent to the concept of tolerance, encapsulated in the idea that ‘one cannot tolerate ideas of which one approves’ (Gibson, 2006, p. 22). In this view, tolerance is conceptualized as an attitude of accommodation despite disapproval, a form of forbearance or endurance of something one does not necessarily like (Cohen, 2004; Gibson, 2006; Verkuyten et al., 2023). Whilst little is known as to what causes one to feel tolerated, the experience of tolerance is shaped by perceptions of implicit devaluation and disapproval from the dominant group, where the non‐dominant group occupies a position of relative moral inferiority (Insel, 2019), coupled with an attitude and expectation of non‐interference from the dominant group. This non‐interference can be further seen as legitimizing the power differences between the dominant and non‐dominant group and reinforcing the dominance of those who tolerate (Verkuyten et al., 2020). Tolerance represents an intermediate stance, neither full acceptance and inclusion nor complete exclusion or rejection (Cvetkovska et al., 2020, 2021; Scanlon, 2003; Verkuyten et al., 2019).

Whilst definitions of tolerance vary across disciplines, research in social and intercultural psychology commonly adopts conceptualizations that emphasize tolerance as enduring or ‘putting up with’ differences (Cvetkovska et al., 2020; Verkuyten et al., 2023). For instance, a recent free‐response survey study found that nearly three‐quarters of participants described tolerance as enduring beliefs or behaviours they disapproved of rather than as genuine openness (Verkuyten & Kollar, 2021). Based on this, we define tolerance as forbearance of beliefs, behaviours, identities or opinions that one does not necessarily agree with or approve of, a definition closer to a permission‐based rather than respect‐based conception of this construct (see Forst, 2018).

Although tolerance may be seen as a positive attribute from the perspective of the *tolerator*, research focussing on the experiences of those being *tolerated* paints a more complex picture. Minoritized individuals who feel tolerated sometimes experience threats to their social identity needs. For instance, ethnic minorities who feel tolerated report lower well‐being and reduced national identification compared to those who feel accepted (Verkuyten et al., 2019, 2020). Furthermore, perceptions of being tolerated can undermine an individual's sense of self‐esteem, meaning, belonging and efficacy, leading to negative outcomes such as reduced self‐worth, increased anxiety and depressive symptoms and lower life satisfaction (Bagci et al., 2020). Nevertheless, feeling tolerated is associated with better well‐being and more optimistic expectations of future treatment than outright rejection or discrimination (Adelman et al., 2023, 2024; Cvetkovska et al., 2020, 2021).

In summary, despite the promotion of tolerance as a key to managing differences, research shows that for those on the receiving end, it can feel ambivalent or, at times, even alienating. It occupies a space between exclusion and inclusion; less harmful than outright rejection but falling short of genuine acceptance and belonging. In the workplace, this nuance becomes especially critical. Organizations increasingly frame diversity not merely as a fact to be managed but as a strategic asset that is central to innovation, collaboration and performance. In such settings, where employees with diverse identities work in close, interdependent relationships, mere tolerance may prove insufficient. Although preferable to discrimination, a culture of tolerance may still limit the full expression and contribution of minoritized employees. The next section explores how perceptions of tolerance may manifest in workplace contexts and why they matter for the lived experiences of LGBTQ+ employees.

### Tolerance in the workplace

Though tolerance may be regarded as essential for peaceful coexistence in society (UN, 1945), in the workplace, coexistence is not the end goal. Instead, organizations emphasize collaboration, mutual reliance and the productive exchange of ideas. Despite this, little is known about which factors in interdependent professional settings engender tolerance.

Diverse workforces are often promoted as offering a competitive advantage by bringing together a range of perspectives that can drive innovation, improve decision‐making and enhance organizational outcomes (Green et al., 1969; Gröschl, 2011; Phillips & O'Reilly, 1998; Shore et al., 2018; Wentling & Palma‐Rivas, 1998). However, these benefits rely on an important assumption, that is, diverse viewpoints are not only present but also genuinely valued. If diversity is merely tolerated rather than actively embraced, the full potential of these varied perspectives may not be realized.

Research has shown that perceptions of inclusion and acceptance are critical for minoritized employees to feel psychologically safe and empowered to share their insights (Li et al., 2019; Mor Barak, 2015; Mor Barak et al., 2016). In contrast, environments where individuals feel merely tolerated may suppress openness and reduce engagement. Recent findings suggest that tolerance can discourage individuals from expressing dissenting views or challenging dominant norms (Adelman et al., 2023), which undermines the very diversity of thought that organizations seek to harness. Furthermore, because tolerance, unlike clear exclusion or discrimination, has an ambiguous nature, it may not prompt a desire to change a potentially suboptimal or even unfair work environment and can instead serve to maintain the status quo. This raises important questions about how tolerance, as a workplace dynamic, may influence the experiences of minoritized groups, particularly those whose identities are less visible.

### LGBTQ+ individuals and tolerance

Although research has begun to uncover the negative implications of being tolerated (Adelman et al., 2023; Bagci et al., 2020; Cvetkovska et al., 2020, 2021), these effects may be particularly acute for individuals with concealable minoritized identities, such as LGBTQ+ employees. For these groups, the message of partial inclusion combined with implicit or explicit discouragement of deviation can create an especially fraught environment. In such contexts, individuals may feel compelled to hide or downplay aspects of their identity to avoid disapproval, judgment or marginalization (Capell et al., 2018; Cipollina & Sanchez, 2022; Follmer et al., 2020; Griffith & Hebl, 2002).

However, identity concealment is associated with a range of negative outcomes, including reduced psychological well‐being (Barreto et al., 2006; Ellemers & Barreto, 2006; Goh et al., 2019; Le Forestier et al., 2022), lower levels of belonging (Newheiser et al., 2017; Newheiser & Barreto, 2014) and diminished job performance (Powers & Ellis, 2014). These risks are further compounded by the persistence of negative social attitudes towards LGBTQ+ individuals, both inside and outside of the workplace (Coffman et al., 2017; European Union Agency for Fundamental Rights, 2024).

Moreover, LGBTQ+ identities are often entangled with moral and religious discourses (Embrick et al., 2007; Hebl et al., 2014), which can heighten their vulnerability in workplace cultures that emphasize tolerance. When identities are viewed through a moral lens—particularly as ‘wrong’ or ‘sinful’, they are more likely to evoke swift judgement and disapproval (Verkuyten et al., 2019, 2023). In workplaces where tolerance is the dominant norm, such moralized views may be implicitly permitted, leading LGBTQ+ employees to feel both hyper visible and unsupported. These dynamics underscore the precarious position of LGBTQ+ employees in environments that prioritize tolerance over inclusion.

### Diversity approaches as signals of being tolerated

Recent literature increasingly emphasizes leveraging diversity approaches to cultivate safe and inclusive work environments (Bell et al., 2011; Li et al., 2019; Mor Barak et al., 2016), particularly for minoritized groups such as racial minorities (for a review, see Gündemir et al., 2019). This research shows that identity‐conscious approaches can decrease prejudiced beliefs (Plaut et al., 2018; though see Wolsko et al., 2000 for contrasting effects on stereotyping), increase perspective‐taking (Sparkman et al., 2019) and enhance self‐esteem, work engagement and job satisfaction amongst minoritized employees (for a meta‐analysis see Leslie et al., 2020).

Whilst research has highlighted how identity‐conscious versus identity‐blind approaches signal in/exclusion, it has yet to address their potential connection to perceptions of being tolerated. Emerging evidence suggests that identity‐blind strategies may inadvertently produce negative outcomes for minoritized groups, including reduced perceptions of support, diminished identity safety and increased concealment of identity (Kirby, Barreto, et al., 2023; Mor et al., 2024; Pichler et al., 2017; Schönauer et al., 2025). We argue that such strategies may un‐intentionally foster a sense of being merely tolerated. When diversity strategies de‐emphasize or avoid recognizing minoritized identities, individuals may perceive this as a lack of appreciation for their identity This may then lead to feelings of being merely tolerated rather than genuinely valued. Relatedly, scholars argue that incorporating tolerance into diversity and inclusion (D&I) practices, such as diversity training, risks fostering environments where individuals are conditionally accepted rather than fully valued (Gebert et al., 2017; Lozano & Escrich, 2017).

We thus propose that the de‐emphasis of social group differences in identity‐blind approaches is interpreted by LGBTQ+ employees as a signal of a tolerant environment. Testing this link is crucial, given prior research showing that LGBTQ+ employees are particularly sensitive to diversity approaches when assessing how safe it is to be their authentic selves, feel a sense of belonging and decide whether to disclose their identity (Cipollina & Sanchez, 2022; Howansky et al., 2021; Kirby, Barreto, et al., 2023; Mor et al., 2024). Importantly, research has shown that these assessments influence LGBTQ+ employees' attraction to employers *and* their intentions to remain with an organization (Mor et al., 2024).

If identity blindness is found to contribute to perceptions of being tolerated amongst (prospective) LGBTQ+ employees, it would suggest that the impact of diversity approaches extends beyond shaping career decisions to impacting day‐to‐day employee collaboration and interaction. Such perceptions could stifle the effective exchange of diverse perspectives (Adelman et al., 2023)—an essential driver of organizational outcomes like innovation. Hence, our first research question is whether LGBTQ+ individuals associate different diversity approaches, particularly identity blindness, with perceptions of being tolerated.

### Additional factors of interest

In addition to exploring the relationship between diversity approaches and perceptions of being tolerated, we seek to examine two additional factors that may influence the direction or strength of this relationship: multiple minoritized group membership and the perceived diversity approach of one's leader.

#### Multiple minoritized group membership

Tolerance, when defined as forbearance of beliefs, practices or identities one disagrees or disapproves of, reflects an unequal power dynamic between those who tolerate and those who are tolerated. Such power imbalances are particularly impactful for individuals who are further removed from the dominant culture (Cvetkovska et al., 2020; Verkuyten et al., 2023). Indeed, research on intersectionality has widely documented the compounded and unique challenges that individuals with multiple marginalized identities face in gaining acceptance and recognition (McCluney & Rabelo, 2019; Purdie‐Vaughns & Eibach, 2008). This suggests that LGBTQ+ individuals who also belong to other minoritized or marginalized groups may be especially vigilant to tolerance cues inferred from diversity approaches (Chaney et al., 2021; Emerson & Murphy, 2014; Hollis, 2022; Kruk & Matsick, 2021; Settles & Buchanan, 2014).

Although many different intersections can be explored, this study specifically focusses on race/ethnicity and transgender identity. First, racial or ethnic group membership continues to be a significant source of bias and discrimination in the workplace (Salari et al., 2024; Whitaker, 2019). LGBTQ+ people of colour may experience compounded bias, facing discrimination based on both their sexual/gender orientation *and* racial/ethnic identity. Second, transgender people within the LGBTQ+ umbrella, particularly those who also belong to a sexual minority face some of the most severe challenges (Cech & Rothwell, 2020; McFadden & Crowley‐Henry, 2016; Pepper & Lorah, 2008). Additionally, in many countries anti‐trans sentiment is on the rise, even surpassing anti‐gay sentiments (Cancela et al., 2024; European Union Agency for Fundamental Rights, 2024; Napier, 2024), further exacerbating their exposure to bias and prejudice. Both LGTBQ+ people of colour and transgender individuals thus are likely to have heightened sensitivity to cues signalling acceptance or the lack thereof due to their frequent encounters with both anti‐LGBTQ+ and racial/ethnic bias (Cech & Rothwell, 2020; Emerson & Murphy, 2014; Hollis, 2022). Thus, we examine whether and how these intersecting identities influence the inferences LGBTQ+ individuals draw about tolerance derived from organizational diversity approaches.

#### Perceived diversity approach of leaders

The role of diversity approaches in shaping an organization's climate is well established. These approaches are often conveyed at an organizational level through formal policies and vision statements, signalling the blueprint organizations follow towards inclusion (Leslie & Flynn, 2022; Li et al., 2019; Wu & Apfelbaum, 2024). However, organizational climate is also critically influenced by leadership. Leaders—such as supervisors and managers—not only directly shape their teams’ behaviours and experiences but also serve as representatives of organizational values. Through their actions, leaders establish norms and expectations and set the tone for appropriate conduct (Grojean et al., 2004; İşçi et al., 2015; Koene et al., 2002).

The diversity approach modelled by leaders significantly impacts employees’ perceptions of the organizational climate (Dang et al., 2022; Dwertmann & van Dijk, 2021; Homan et al., 2020; Lee et al., 2021), making it a critical factor in shaping perceptions of tolerance amongst LGBTQ+ employees. Leaders influence these perceptions both through direct interactions with employees and by modelling inclusive or exclusive behaviours. Additionally, as representatives of the organization, leaders serve as a link between the organization's broader values and individual employee experiences. This dual influence suggests that the diversity approach communicated by leaders may play two key roles. First, the perceived diversity approach of leaders may be directly associated with LGBTQ+ employees’ perceptions of tolerance. Second, leaders’ approaches may moderate the impact of the organization's overall diversity approach on these perceptions, either amplifying or mitigating its effects.

## OVERVIEW OF STUDIES

We explored the role of diversity approaches in shaping perceptions of tolerance in the workplace among LGBTQ+ individuals across two studies. The data were collected as part of Mor et al. (2024) research on diversity approaches and LGBTQ+ workers. For the current analyses, we focus specifically on findings related to perceptions of tolerance included in the studies for exploratory purposes. The relevant pre‐registrations detailing this exploratory measure are publicly available (https://osf.io/my2zr/?view_only=6c5ed5842df744b8a4dd957dea8e382f). The studies were programmed in Qualtrics and shared via the Prolific crowdsourcing platform. Participants were selected based on the following inclusion criteria: (1) participants had to be adults (18 years or older), (2) participants were required to reside in the United Kingdom and (3) participants needed to self‐identify as lesbian, gay, bisexual, transgender or otherwise queer (LGBTQ+). Participants who identified as both cisgender and heterosexual, as well as those with incomplete data, were excluded from the analysis. Additionally, ethnicity data provided by Prolific were incorporated to assess potential intersectional factors in the findings. Unless otherwise specified, responses were collected using a seven‐point Likert scale (1 = *Strongly disagree* to 7 = *Strongly agree*). Upon completing the study, participants were fully debriefed and given an opportunity to provide feedback.

## STUDY 1

### Participants and procedure

The study uses an available dataset collected for Mor et al. (2024), including 499 responses. Thirty‐seven participants did not meet our pre‐registered criteria and were excluded before analysis. The final sample included 462 participants (*M*_age_ = 30.48, *SD*_age_ = 10.02). A post‐hoc sensitivity analysis of the collected sample indicated sufficient power (1 − *β* = 0.80, *α* = 0.05) to detect an effect size of *d* = 0.26 (Faul et al., 2007). Full breakdown of the sample characteristics can be found in Table 1.

**TABLE 1:** Demographic composition of participant samples of Studies 1and 2.

Demographic characteristics	Study 1		Study 2	
	*n*	%	*n*	%
Work status				
Employed full‐time	217	47	333	74.8
Employed part‐time	90	19.5	112	25.2
Unemployed looking for work	22	4.8	–	–
Unemployed not looking for work	46	10	–	–
Retired	6	1.3	–	–
Student	81	17.5	–	–
Gender group				
Male	151	32.7	141	31.7
Female	234	50.6	237	53.3
Non‐binary	60	13	51	11.5
Genderfluid	8	1.7	6	1.3
Agender	4	0.8	4	0.9
Self‐described	5	1.1	6	1.3
Transgender identity				
Yes	112	24.2	102	22.9
No	349	75.5	343	77.1
Other	1	0.2	0	0
Sexual orientation				
Gay	66	14.3	70	15.7
Lesbian	46	10	80	18
Bisexual	212	45.9	195	43.8
Queer	36	7.8	26	5.8
Asexual	40	8.7	25	5.6
Pansexual	58	12.6	42	9.4
Heterosexual	4	0.9	5	1.1
Self‐described	0	0	2	0.4
Ethnicity				
White	416	90	398	89.4
Black	9	1.9	7	1.6
Asian	15	3.2	15	3.4
Mixed	18	3.9	19	4.3
Other	2	.4	3	0.7
Missing/unknown	2	.4	3	0.7

After providing informed consent, participants were randomly assigned to either an identity‐blind condition or an identity‐conscious condition. They were then presented with the diversity mission statement of a fictitious organization and asked to form an impression based on the limited information. Participants rated their perceptions of the statement, including perceived tolerance, and assessed the organization's attractiveness. Finally, participants completed demographic questions. Full materials can be found in the supporting information.

### Materials and measures

Depending on the assigned condition, participants viewed a version of a webpage from a fictional organization, Wynn Inc., which featured a diversity mission statement (texts are modelled after Purdie‐Vaughns and colleagues, Purdie‐Vaughns & Eibach, 2008; see Mor et al., 2024). The mission statement in the identity‐blind condition emphasized ignoring differences and fostering equality through a focus on similarities. A sample phrase is ‘*While other firms mistakenly focus on their staff's diversity, we at Wynn Inc. train our workforce to embrace their similarities*.’ In contrast, the identity‐conscious condition highlighted the importance of diversity and encouraged recognizing and valuing differences, including phrases such as ‘*While other firms mistakenly try to shape their staff into a single mold, we at Wynn Inc. believe that embracing our differences enriches our culture*.’

Anticipated tolerance was measured with one item adapted from Cvetkovska et al. (2021). Participants rated the statement ‘I anticipate being tolerated at this company, meaning that people will not really approve of my identity, but rather will endure and put up with me at work if I worked at Wynn Inc.’

We further also collected demographic data, including the participants’ age, employment status, gender identity and sexual orientation. Open‐response entries for gender identity and sexual orientation (‘I prefer to self‐describe’) were reviewed and recategorized as needed for analysis.

## RESULTS

### Descriptives and correlations

Across our sample, anticipated tolerance had a mean around the midpoint of the scale (*M =* 3.77, *SD =* 1.90). Table 2 presents descriptive statistics and intercorrelations for both the primary variables of the current study. The full correlation matrix with all the key variables from Mor et al. (2024), from which the current dataset was derived, can be found in the supporting information.

**TABLE 2:** Descriptive statistics and bivariate correlations of Studies 1 and 2.

Variable	*M* (SD)/*N* (%)	1	2	3	4	5
Study 1						
1. Age	30.48 (10.02)	–				
2. Identity‐conscious condition	228 (49.4%)	.12*	–			
3. Transgender identity	111 (24%)	−.09^+^	.01	–		
4. Racial/ethnic minority identity	44 (9.5%)	−.11*	−.04	.01	–	
5. Anticipated tolerance	3.77 (1.90)	−.18**	−.26**	.17**	.10*	
Study 2						
1. Age	32.73 (9.41)	–				
2. Organizational diversity approach	4.87 (1.37)	.02	–			
3. Leader diversity approach	4.73 (1.35)	−.03	.67**			
4. Transgender identity	102 (22.9%)	−.25**	−.15**	−.11*		
5. Racial/ethnic minority identity	44 (9.9%)	−.06	−.07	.00	−.05	
6. Perceived tolerance	2.65 (1.62)	−.16**	−.41**	−.39**	.26**	.07

*Note*: *N*_Study 1_ = 462; *N*_Study 2_ = 445.

^+^ *p* < .10;

* *p* < .05;

** *p* < .01.

### Effect of the diversity approach on anticipated tolerance

An independent sample *t*‐test indicated that participants in the identity‐blind condition anticipated higher tolerance (*M* = 4.26, *SD* = 1.85) at Wynn Inc. compared to participants in the identity‐conscious condition (*M* = 3.27, *SD* = 1.80; *t*[460] = 5.76, *p* < .001, 95% CI [0.65, 1.32], Cohen's *d* = 1.84).

Further, we find that in the identity‐conscious condition, tolerance is significantly lower than the mid‐point of the scale (*t*[227] = −6.06, *p* < .001, 95% CI [−0.96, −0.49], Cohen's *d* = 1.81), and in the identity‐blind condition, tolerance is significantly higher than the mid‐point of the scale (*t*[233] = 2.11, *p* = .036, 95% CI [0.02, 0.50], Cohen's *d* = 1.86).

### Multiple group membership: Transgender identity

We performed step‐wise regression analyses to explore (a) possible differences between cisgender (coded as 0) and transgender (coded as 1) participants in their anticipated tolerance and (b) a possible interaction effect between the organizational approach and gender identity.

Across conditions, transgender participants reported significantly higher anticipated tolerance (*b* = 0.71, *SE* = 0.20, *p* = .001, 95% CI [0.32, 1.10]) than cisgender participants. We found no support for an interaction between organizational approach and gender identity on the dependent measures (*b* = −0.30, *SE* = 0.40, *p* = .447, 95% CI [−1.08, 0.47]).

### Multiple group membership: Race/ethnicity

We performed step‐wise regression analyses to explore (a) possible differences between white (coded as 0) and non‐white (coded as 1) participants in their reported anticipated tolerance and (b) a possible interaction effect between the organizational approach and race/ethnicity.

Across conditions, non‐white participants reported significantly higher anticipated tolerance (*b* = 0.61, *SE* = 0.30, *p* = .037, 95% CI [0.04, 1.17]) than white participants. We found no support for an interaction between the organizational approach and race/ethnicity on the dependent measures (*b* = 0.37, *SE* = 0.58, *p* = .526, 95% CI [−0.78, 1.52]).

### Discussion

Our findings suggest that LGBTQ+ individuals are more likely to anticipate a tolerant environment in organizations that employ an identity‐blind rather than an identity‐conscious approach in their messaging. Consistent with our expectations, the negative effects of marginalization are compounded for individuals with intersecting marginalized identities (Chaney et al., 2021; Purdie‐Vaughns & Eibach, 2008; Settles & Buchanan, 2014). Within our LGBTQ+ sample, transgender participants and non‐white participants anticipated higher levels of tolerance compared to their cis‐gender and white counterparts, highlighting the heightened sensitivity to marginalization cues amongst those with multiple marginalized identities. However, we find no evidence that responses to diversity approaches in terms of tolerance differ based on multiple marginalized identities.

## STUDY 2

### Participants and procedure

The study uses an available dataset we collected for Mor et al. (2024), including 500 responses. Fifty‐five participants did not meet our pre‐registered inclusion criteria and were excluded before analysis. The final sample included 445 participants (*M*_age_ = 32.73, *SD*_age_ = 9.41). A post‐hoc sensitivity analysis of the collected sample indicated sufficient power (1 − *β* = 0.80, *α* = 0.05) to detect an effect size of *f* ^2^ = 0.02 (Faul et al., 2007). Full breakdown of the sample characteristics can be found in Table 1.

The study was advertised as an investigation into employees’ workplace experiences. After providing informed consent, participants completed a series of demographic questions, including age, gender identity and sexual orientation. Following this, participants were asked a range of questions about their workplace, focussing on their perceptions and experiences as employees. These questions covered topics such as the diversity approach of their current organization, the diversity approach of their direct supervisor, their intentions to leave the organization (turnover intentions) and the sense of being tolerated at work. The same demographic information as collected in Study 1 was also collected here.

### Measures

*Organizational diversity approach* was measured with nine items: four items assessing perceived identity‐consciousness of own organization and five items assessing perceived identity‐blindness, adapted from Dang et al. (2022). Example items include ‘My organization behaves in ways that ignore employees' demographic background.’ and ‘My organization believes that employees' demographic differences should be acknowledged and valued.’ A principal component analysis (PCA) was conducted on the items using a direct oblimin rotation (Conway & Huffcutt, 2003), and the details of the PCA are available in Mor et al. (2024). Following the pre‐registered approach and consistent with theoretical and empirical support for treating ideology as a unitary construct (Koenig & Richeson, 2010; Martin & Phillips, 2017), a single measure of perceived diversity approach was created. Higher scores on this measure indicate a more conscious approach, whilst lower scores reflect a more blind approach (*α* = .90).

*The leader diversity approach* was measured using a similar scale to the organizational diversity approach scale, with nine items: four assesing percieved identity‐consciousness five assesing percieved identity‐blindness. Participants were asked to think about their direct supervisor or the one they interact with most if they had multiple (Dang et al., 2022). A principal components analysis (PCA), using a direct oblimin rotation (Conway & Huffcutt, 2003), yielded two factors with eigen values of 5.16 (four identity consciousness items and three reverse coded blindness items accounting for 57.40% of the variance) and 1.42 (two blindness items accounting for 15.76% of the variance). Compared to the organizational diversity approach scale, the leader diversity approach scale had an additional item that loaded on the first factor . However, for consistency, we created a unitary construct using the same items as the ones we used for the organizational diversity approach (*α* = .91).

*Tolerance* was measured using a single item adapted from Cvetkovska et al. (2021). Participants rated the statement ‘I experience being tolerated at my organization, meaning that people don't really approve of my identity, but rather endure and put up with me at work.’

Participants reported the number of years they had been working at their current place of work. Due to the nature of responses (e.g., including ‘years’ or ‘half’), this variable had to be manually corrected and transformed into a numerical variable in the statistical software program IBM SPSS (Version 27).

## RESULTS

### CMB correction

Following scholarly recommendations to mitigate the risks associated with common method bias (CMB)—artificially inflated relationships between variables (Spector & Brannick, 2010)—we included a marker variable in the survey that was theoretically unrelated to any of our other variables of interest (Simmering et al., 2015; see also Mor et al., 2024). This variable, namely, the preference for colour green, was then controlled for in our analyses. Inclusion of this variable had no notable effect on our findings.

### Descriptives and correlations

Across our sample, tolerance had a mean smaller than the midpoint of the scale (*M =* 2.65, *SD =* 1.62). Table 2 presents descriptive statistics and intercorrelations for both the primary variables of the current study. The full correlation matrix with all the key variables from Mor et al. (2024), from which the current dataset was derived, can be found in the supporting information.

### Effect of the organizational diversity approach on tolerance

We performed a linear regression to explore the effect of the organizational diversity approach on perceived tolerance. We found that higher perceived organizational identity‐consciousness was related to lower tolerance (*b* = −0.49, *SE* = 0.05, *p* < .001, 95% CI [−0.59, −0.39]).

### Multiple group membership: Transgender identity

We performed step‐wise regression analyses to explore (a) possible differences between cisgender (coded as 0) and transgender (coded as 1) participants in their reported tolerance and (b) a possible interaction effect between the organizational approach and gender identity.

Across conditions, transgender participants reported significantly higher perceived tolerance (*b* = 0.79, *SE* = 0.19, *p* < .001, 95% CI [0.46, 1.11]) than cisgender participants. We obtained no support for an interaction between the organizational approach and gender identity on perceived tolerance (*b* = −0.01, *SE* = 0.15, *p* = .939, 95% CI [−0.29, 0.31]).

### Multiple group membership: Race/ethnicity

We performed a series of regression analyses to explore (a) possible differences between white (coded as 0) and non‐white (coded as 1) participants in their reported tolerance and (b) a possible interaction effect between the organizational approach and race/ethnicity. We obtained no main or interactive effects of race/ethnicity on perceived tolerance.

### Effect of the leader diversity approach on tolerance

We performed a linear regression to explore the effect of the leader diversity approach on perceived tolerance. We found that higher perceived leader identity‐consciousness was related to lower perceived tolerance (*b* = −0.46, *SE* = 0.05, *p* < .001, 95% CI [−0.57, −0.36]).

### Interaction between the organization and leader diversity approaches on tolerance

Finally, we aimed to explore whether organizational and leader diversity approaches were independently associated with perceived tolerance, as well as how the leader diversity approach might moderate the effect of the organizational diversity approach on tolerance. To address these questions, we performed stepwise linear regressions, with the main effects added in step one and the interaction effect added thereafter. In these models, we included both organizational and leader diversity approaches as independent variables and then added an interaction term between the two.

**FIGURE 1:**
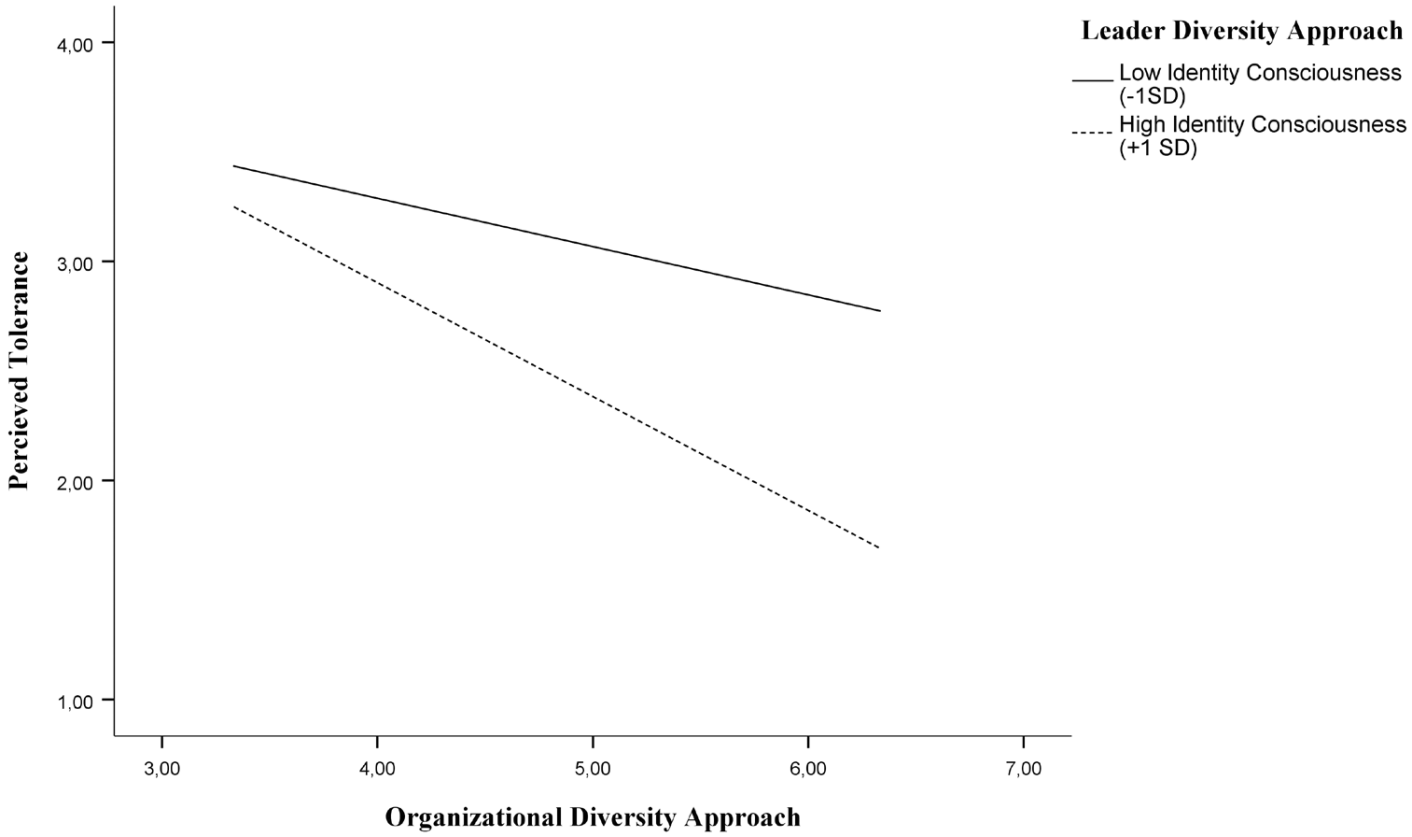
Moderation effect of the perceived organizational diversity approach on perceived tolerance by the perceived leader diversity approach in Study 2.

We found that higher perceived organizational identity‐consciousness (*b* = −0.33, *SE* = 0.07, *p* < .001, 95% CI [−0.47, −0.20]) and higher perceived leader identity consciousness (*b* = −0.24, *SE* = 0.07, *p* < .001, 95% CI [−0.37, −0.10]) were both significantly associated with lower perceived tolerance. Additionally, the interaction between organizational and leader diversity approaches also had a significant effect on perceived tolerance (*b*_interaction_ = −0.11, *SE* = 0.03, *p* = .002, 95% CI [−0.17, −0.04]). An examination of the simple slopes indicated that when participants perceived their organization as relatively lower on identity‐consciousness (−1 *SD*), perceived leader identity‐consciousness was not significantly associated with perceived tolerance (*b* = −0.07, *SE* = 0.09, *p* = .457, 95% CI [−0.24, 0.11]); however, when participants perceived their organization as relatively higher on identity‐consciousness (+1 *SD*), perceived leader identity‐consciousness was associated with significantly reduced perceived tolerance (*b* = −0.38, *SE* = 0.08, *p* < .001, 95% CI [−0.55, −0.22]). For the visualization of this interaction, see Figure 1.

### Discussion

Study 2 builds upon and reinforces the findings of Study 1. Specifically, it demonstrates that an identity‐blind diversity approach not only signals tolerance to LGBTQ+ individuals evaluating organizations from the outside but is also associated with LGBTQ+ employees within such workplaces perceiving higher levels of tolerance. In alignment with Study 1, transgender employees reported higher levels of tolerance compared to their cis‐gender counterparts. However, unlike Study 1, no significant effects of race/ethnicity on perceived tolerance were observed in this study. Additionally, we found that the link between diversity approaches and perceptions of tolerance is evident not only in the organization's formal communications but also in the diversity approaches adopted by leaders. The diversity approach endorsed and modelled by leaders has an influence on LGBTQ+ employees' perceptions of tolerance, highlighting the critical role of leadership in shaping workplace climate. The diversity approach adopted by leaders was independently related to perceptions of tolerance, distinct from the impact of the organizational diversity approach. Importantly, leader diversity approaches also moderated the relationship between organizational diversity approaches and perceived tolerance. Specifically, in organizations perceived as endorsing an identity‐conscious approach, leaders who also endorsed an identity‐conscious approach further reduced perceptions of tolerance amongst LGBTQ+ employees, arguably fostering a more inclusive environment. These findings underscore the critical interplay between organizational and leadership‐level diversity, highlighting the importance of alignment between the two in affecting workplace experiences for LGBTQ+ employees.

## GENERAL DISCUSSION

Diversity approaches are widely adopted in organizations and often guide their actions and initiatives related to diversity issues. In recent decades, the impact of these approaches has received growing scholarly attention, with studies showing that they can influence employee responses across a variety of workplace‐relevant domains, across many social identity groups (e.g., Gündemir et al., 2019; Kirby, Russell Pascual, et al., 2023; Leslie et al., 2020; Martin, 2023; Mor et al., 2024). Whereas previous studies have primarily examined how diversity approaches relate to positive (e.g., belonging) or negative (e.g., prejudice) workplace perceptions, our focus here has been on a more conceptually ambiguous yet highly relevant outcome, that is, tolerance, a complex construct somewhere between inclusion and exclusion. We examined whether, and how, organizational diversity approaches might inadvertently cue perceptions of tolerance rather than genuine inclusion to LGBTQ+ individuals in workplace settings. Our findings reveal that organizations adopting an identity‐blind diversity approach, as opposed to an identity‐conscious one, signal a climate of tolerance to prospective employees and engender perceptions of tolerance amongst current LGBTQ+ employees. Additional findings highlighted that diversity approaches modelled by leaders, including supervisors and managers, can also significantly shape perceptions of tolerance. Specifically, when leaders were perceived to embrace a more identity‐conscious approach, perceptions of tolerance amongst LGBTQ+ employees were reduced. Furthermore, the negative relationship between an identity‐conscious organizational approach and perceived tolerance was amplified when leaders also endorsed identity‐conscious strategies.

### Theoretical implications

This study extends and integrates the diversity approach paradigm with tolerance research, revealing how an identity‐blind approach by de‐emphasizing demographic differences signals tolerance to LGBTQ+ employees. Our work contributes to emerging research on the impact of diversity approaches on employee groups with concealable identities (Kirby, Russell Pascual, et al., 2023; Mor et al., 2024). We identify a novel effect of these diversity strategies on such groups, who may be especially sensitive to cues of tolerance.

Our examination of intersectional dynamics is valuable for untangling the intragroup variation within LGBTQ+ employee populations. First, our study uncovered critical main effects of multiple group memberships. Transgender LGB individuals consistently reported higher anticipated and perceived tolerance than cisgender counterparts, regardless of the diversity approach. For LGBTQ+ people of colour, Study 1 showed heightened anticipated tolerance, but Study 2 found no significant difference in perceived tolerance compared to white LGBTQ+ participants. One explanation for the differences in results is that LGBTQ+ employees of colour may have greater access to community resources, which can buffer against feelings of tolerance and foster inclusion, unlike transgender individuals who may have more limited access. However, when evaluating potential future employers (Study 1), such resources may be less salient, leading to heightened perceptions of anticipated tolerance for both groups. As a result, anticipated tolerance may be more prominent than experienced tolerance for LGBTQ+ employees of colour in their current workplace (Study 2). Second, the absence of interactive effects between transgender or racial identity and organizational diversity approaches on tolerance is intriguing. Whilst the current data do not allow us to draw definitive conclusions about the reasons behind these null effects, one possible explanation is the use of broad and inclusive language (i.e., ‘demographic groups’) in the diversity approach manipulations and measures, which did not explicitly reference specific sub‐identities. This general framing may have shaped participants' perceptions, all of whom identify as members of the LGBTQ+ community. Specifically, the presence or absence of recognition of demographic group membership within the diversity statements or measures may have activated a more encompassing minority group identity amongst participants and, consequently, may not have activated specific facets of their intersectional identities. Whilst this remains a tentative interpretation, it highlights the importance of future iterations of this line of research. For instance, researchers could vary the degree of specificity with which intersectional identities are incorporated into predictor variables, in order to test whether such specificity elicits different response patterns.

We further contribute to the literature by unpacking and highlighting the pivotal role of leaders in signalling tolerance. Leaders act as an embodiment of organizational culture, shaping interpersonal dynamics within their teams and serving as representatives of the organization's values. This dual role positions leaders as critical actors in translating organizational diversity strategies into day‐to‐day experiences for employees (Grojean et al., 2004; Homan et al., 2020; İşçi et al., 2015; Koene et al., 2002). Our results suggest that the diversity approaches modelled by leaders can be as important as those conveyed at the organizational level. Moreover, our findings highlighted the amplifying or mitigating role leaders play in relation to organizational diversity strategies. Within organizations that were perceived as more identity‐conscious, leaders who similarly adopted identity‐conscious practices further diminished perceptions of tolerance, creating a synergistic effect that reinforced the perceived climate for inclusion. The alignment between organizational and leadership‐level diversity strategies emphasizes the critical importance of consistency across hierarchical levels in the workplace (Nishii et al., 2018; Wright & Nishii, 2004). Such alignment not only promotes a more inclusive environment but also reduces perceptions of mere tolerance. By extending the paradigm of diversity approaches, this research demonstrates how these strategies function across multiple levels—organizational and leadership—and interact in complex ways to shape employee perceptions.

### Practical implications

Diversity is not only a characteristic of most modern workplaces but also frequently regarded as a strategic asset, capable of driving innovation, enhancing decision‐making and fostering overall organizational success (Green et al., 1969; Gröschl, 2011; Shore et al., 2018; Wentling & Palma‐Rivas, 1998). However, the effectiveness of this diversity advantage depends on an inclusive environment where diverse individuals feel valued and empowered to share their unique perspectives. The results of our studies suggest that identity‐blind diversity approaches, which de‐emphasize demographic differences, contribute to perceptions of being merely tolerated rather than fully accepted for LGBTQ+ individuals. A tolerance‐focussed climate may undermine the collaborative and innovative potential of diverse teams, as employees who feel tolerated may be less likely to voice unique ideas, perspectives or dissenting opinions (Adelman et al., 2023). This stifling of open dialogue compromises an organization's ability to capitalize on the diversity of its workforce. Therefore, organizations should be aware of the risks attached to incorporating tolerance into their diversity strategies and instead consider creating an environment that fosters inclusion and acceptance, which can be realized by employing an identity‐conscious diversity approach (Kirby, Russell Pascual, et al., 2023; Mor et al., 2024).

In addition, our results underscore the importance of alignment between organizational messaging and leadership behaviour. Organizations should ensure that their formal diversity policies and vision statements are mirrored in the actions and attitudes of their leadership. Alignment between organizational and leader diversity approaches can amplify the effects of an identity‐conscious approach in reducing perceptions of tolerance for LGBTQ+ employees. By adopting identity‐conscious diversity approaches at both organizational and leadership levels, organizations can not only mitigate the negative consequences of tolerance but potentially enhance the innovative and collaborative potential of their diverse workforce.

### Limitations

This research is not without its limitations, which should be considered when interpreting the findings and designing future studies. Our focus on researching LGBTQ+ employees led to not recruiting a very ethnically diverse sample, with only ~10% of the sample being non‐white, which limits our ability to draw strong conclusions on possible intersectional struggle faced by individuals with multiple marginalized identities. Furthermore, our focus on LGBTQ+ individuals as a group that responds in particular ways to organizational cues may have un‐intentionally suggested that we view this group as a monolith, overlooking individual differences. It is important to clarify that our aim is to advance theory by addressing group‐based struggles and challenges that persist in both society and the workplace. At the same time, we recognize the importance of considering a variety of individual differences that may interact with group identity to shape responses to diversity approaches (e.g., Kirby & Kaiser, 2020).

Additionally, our study measured tolerance using a single item adapted from prior work (Cvetkovska et al., 2020). Whilst this measure captures a core component and widely studied aspect of tolerance, the single‐item measure limits our ability to assess its potential multi‐dimensionality. For example, tolerance may involve varying experiences such as conditional acceptance, covert prejudice or subtle exclusion, each of which may be differentially influenced by diversity approaches. Furthermore, one may question whether our operationalization of tolerance as perceived forbearance with one's identity may conceptually overlap with our measure of identity blindness, which assesses the extent to which employees perceive their organization as downplaying demographic differences. This overlap could contribute to inflated correlations between constructs and should be carefully considered by scholars studying diversity approaches in conjunction with tolerance. It is, however, important to recognize distinctions between these measures in the context of the current contribution. Diversity approaches represent espoused or enacted, prescriptive frameworks on how to engage with diversity (Wu & Apfelbaum, 2024), whereas tolerance reflects employees' evaluative psychological experience of these approaches. In other words, in our studies, diversity approaches describe policy orientation that is external to the participant and tolerance refers to a personal interpretation of its impact. This distinction is particularly evident in our experimental study, where the causal relationship between the approach and anticipated tolerance is examined, and participants are randomly assigned to different informational primes (and with no other information) and report varying levels of anticipated tolerance in response. Moreover, whilst high co‐variance between blindness and tolerance could pose a greater threat to our correlational study, we observe only a moderate negative correlation between the two constructs (*r* = −.41). For comparison, we find a much stronger correlation between perceived organizational and supervisory diversity approaches (*r* = .67), suggesting that participants are capable of discerning overlap between constructs when such overlap is present. Furthermore, the moderately high conceptual overlap between identity‐blindness and tolerance could be of theoretical significance and may inspire future research. Specifically, like identity‐consciousness, identity‐blindness represents an approach aimed at promoting cohesion and collaboration in the workplace. However, if this approach un‐intentionally signals ‘endurance without acceptance’ towards individuals with marginalized identities (Leslie, 2019), it could reveal another psychological process explaining why minoritized groups tend to respond less positively to identity‐blindness than to identity‐conscious approaches.

Finally, it is worth considering the measures and manipulations of diversity approaches at a more fundamental level, specifically, along a valence dimension. For example, one may argue that identity blindness is often operationalized in negatively valenced terms, whereas identity consciousness is framed more positively. Although both approaches theoretically aim to improve intergroup relations, these differences in valence may confound research findings. From this perspective, one might expect measures of these approaches to be strongly negatively correlated. However, empirical evidence often contradicts this expectation; several studies report a positive association between measures of identity conscious approaches (e.g., multi‐culturalism) and identity blind ones (e.g., colour‐blindness ideology; e.g., Wollast et al., 2023). Nonetheless, it is important to acknowledge valence‐related threats to validity to enhance clarity in this research area. Future studies should carefully consider the role of valence, as doing so will enable a more precise delineation of the scope and robustness of diversity approach effects.

### Conclusion

In conclusion, our research highlights that identity‐blind diversity approaches in the workplace can signal a sense of being tolerated rather than truly accepted for LGBTQ+ employees. These findings highlight the importance of recognizing that diversity efforts that overlook or undervalue group‐based differences—such as identity‐blind vision statements—can (un‐intentionally) convey mere tolerance than true acceptance. This approach may have negative consequences for underrepresented groups, including LGBTQ+ employees, and hinder open and safe collaborative environments necessary for the potential benefits of diversity to emerge. Moreover, aligning organizational diversity strategies with inclusive leadership behaviours can enhance support for diverse employees, foster their well‐being and create conditions that allow their unique perspectives to meaningfully contribute to organizational success.

## AUTHOR CONTRIBUTIONS

**Kshitij Mor:** Conceptualization; methodology; formal analysis; visualization; writing – review and editing; writing – original draft; investigation; validation; data curation. **Seval Gündemir:** Conceptualization; writing – review and editing; methodology; supervision; project administration; validation. **Jojanneke van der Toorn:** Conceptualization; writing – review and editing; methodology; validation; project administration; supervision; funding acquisition.

## CONFLICT OF INTEREST STATEMENT

The authors have no known conflict of interest to disclose.

## Supporting information


Table S1.

## Data Availability

The data supporting the findings of this study are openly available on the Open Science Framework (OSF) at https://osf.io/my2zr/?view_only=6c5ed5842df744b8a4dd957dea8e382f. Information about the dataset from which these data were derived is also publicly accessible on OSF at https://osf.io/ysx2w/.

## Footnotes

^1^
Research into diversity approaches has further identified sub‐facets and combinations within organizational diversity strategies that reflect these two overarching ideologies, such as multicultural meritocracy, interculturalism and individual uniqueness. These more specific approaches fall outside the scope of this study but have been discussed in detail by others (Gündemir et al., 2017, 2019; Yogeeswaran et al., 2021).
