# Supplementary material for: ‘Are they just putting up with me’? How diversity approaches impact LGBTQ+ employees' sense of being tolerated at work
Source: Br J Soc Psychol. 2025 Aug 1;64(4):e70006. doi: 10.1111/bjso.70006 (PMC12315620; doi:10.1111/bjso.70006)
Supplement: Supplementary file 1 — Table S1. [file BJSO-64-0-s001.docx]

**Table 1**

*Descriptive Statistics and Correlations of Studies 1 & 2*

| Variable | *M (SD)* | 1 | 2 | 3 | 4 | 5 | 6 | 7 |
| --- | --- | --- | --- | --- | --- | --- | --- | --- |
| *Study 1* |  |  |  |  |  |  |  |  |
| 1. Age | 30.48 (10.02) | - |  |  |  |  |  |  |
| 2. Organizational Attractiveness | 4.76 (1.53) | .12^*^ | - |  |  |  |  |  |
| 3. Anticipated Authenticity | 4.78 (1.89) | .04 | .83^**^ | - |  |  |  |  |
| 4. Anticipated Belonging | 4.94 (1.55) | .05 | .84^**^ | .81^**^ | - |  |  |  |
| 5. Anticipated Justice | 4.97 (1.33) | .09^+^ | .78^**^ | .82^**^ | .81^**^ |  |  |  |
| 6. Anticipated Tolerance | 3.77 (1.90) | -.18^**^ | -.54^**^ | -.57^**^ | -.54^**^ | -.61^**^ |  |  |
| *Study 2* |  |  |  |  |  |  |  |  |
| 1. Age | 32.73 (9.41) | - |  |  |  |  |  |  |
| 2. Organizational Diversity Approach | 4.87 (1.37) | .02 | - |  |  |  |  |  |
| 3. Leader Diversity Approach | 4.73 (1.35) | -.03 | .67^**^ |  |  |  |  |  |
| 4. Turnover Intentions | 3.71 (2.04) | -.16^**^ | -.39^**^ | -.33^**^ |  |  |  |  |
| 5. Perceived Authenticity | 5.12 (1.60) | .12^*^ | .55^**^ | .53^**^ | -.54^**^ |  |  |  |
| 6. Perceived Belonging | 4.79 (1.61) | .15^**^ | .54^**^ | .46^**^ | -.58^**^ | .79^**^ |  |  |
| 7. Perceived Justice | 5.09 (1.46) | .13^**^ | .44^**^ | .42^**^ | -.58^**^ | .71^**^ | .76^**^ |  |
| 8. Perceived Tolerance | 2.65 (1.62) | -.16^**^ | -.41^**^ | -.39^**^ | .46^**^ | -.61^**^ | -.58^**^ | -.54^**^ |

*Note*. *N_Study 1_*= 462; *N_Study 2_* = 445.

^+^ *p*<.10, ^*^*p*<.05, ^**^*p*<.01s
